# Mining the Yucatan Coastal Microbiome for the Identification of Non-Ribosomal Peptides Synthetase (NRPS) Genes

**DOI:** 10.3390/toxins12060349

**Published:** 2020-05-26

**Authors:** Mario Alberto Martínez-Núñez, Zuemy Rodríguez-Escamilla

**Affiliations:** UMDI-Sisal, Facultad de Ciencias, Universidad Nacional Autónoma de México, Puerto de Abrigo s/n, Sisal, Yucatán CP 97355, Mexico

**Keywords:** nonribosomal peptides, metataxonomic, metatranscriptomic, Yucatan coast

## Abstract

Prokaryotes represent a source of both biotechnological and pharmaceutical molecules of importance, such as nonribosomal peptides (NRPs). NRPs are secondary metabolites which their synthesis is independent of ribosomes. Traditionally, obtaining NRPs had focused on organisms from terrestrial environments, but in recent years marine and coastal environments have emerged as an important source for the search and obtaining of nonribosomal compounds. In this study, we carried out a metataxonomic analysis of sediment of the coast of Yucatan in order to evaluate the potential of the microbial communities to contain bacteria involved in the synthesis of NRPs in two sites: one contaminated and the other conserved. As well as a metatranscriptomic analysis to discover nonribosomal peptide synthetases (NRPSs) genes. We found that the phyla with the highest representation of NRPs producing organisms were the Proteobacteria and Firmicutes present in the sediments of the conserved site. Similarly, the metatranscriptomic analysis showed that 52% of the sequences identified as catalytic domains of NRPSs were found in the conserved site sample, mostly (82%) belonging to Proteobacteria and Firmicutes; while the representation of Actinobacteria traditionally described as the major producers of secondary metabolites was low. It is important to highlight the prediction of metabolic pathways for siderophores production, as well as the identification of NRPS’s condensation domain in organisms of the Archaea domain. Because this opens the possibility to the search for new nonribosomal structures in these organisms. This is the first mining study using high throughput sequencing technologies conducted in the sediments of the Yucatan coast to search for bacteria producing NRPs, and genes that encode NRPSs enzymes.

## 1. Introduction

Prokaryotes are the most abundant organisms in coastal and estuarine ecosystems, and their anaerobic respiratory processes contribute to the transformation of nitrogen, sulfur, iron and carbon, playing a key role in the productivity of the coastal marine ecosystem, as well as in the regulation of relevant processes in global biogeochemical cycles [1,2]. The importance of marine microbial diversity not only focuses on their environmental contributions, they also represent a source of both biotechnological and pharmaceutical molecules of importance, such as nonribosomal peptides (NRPs). Soil-inhabiting microorganisms, such as Actinobacteria and Bacilli, and eukaryotic filamentous fungi are mostly producers of NRPs, although marine microorganisms have also emerged as a source for such peptides [3,4,5]. Nonribosomal peptides are secondary metabolites with diverse properties such as toxins, siderophores, pigments, or antibiotics, among others [6]. Until the present, more than 50% of drugs that are in clinical use belong to the NRPs or mixed polyketide-nonribosomal peptide families derived from natural products isolated from marine bacteria. These contribute to 70% of NRPs discovered with the activity of antimicrobial, antiviral, cytostatic, immunosuppressant, antimalarial, antiparasitic, animal growth promoters and natural insecticides [7]. They are synthesized on large nonribosomal peptide synthetase (NRPS) enzyme complexes, which means that their synthesis is independent of ribosomes [8]. The NRPSs are modularly organized, with each module consisting of several domains for the formation of the NRPs such as adenylation (A) domain; peptidyl carrier protein (PCP) or thiolation (T) domain; and condensation (C) domain [9]. The biosynthesis of the NRPs can be carried out by three types of NRPSs: type A, a linear NRPS in which each enzymatic domain is used once during the biosynthesis; type B, an iterative NRPS that uses all its modules more than once during the biosynthesis; whereas Type C is a non-linear NRPS that work more than once during the biosynthesis of a single NRP [10]. These secondary metabolites represent promising scaffolds for the development of new drugs [11] due to the great structural diversity they have, derived from having more than 300 different precursors and are not limited to only 20 proteinogenic amino acids [12]. With the advances of DNA sequencing technologies, today we have an enormous amount of genomes, and even metagenomes, that allow us to experience a renaissance and inaugurate a new era in which we can look for new NRPs [13,14]. Thanks to DNA sequencing of environmental samples, known as metagenomics, it is now possible to get access to functional information of genes that are encoded in genomes of uncultivated bacteria to discover new NRPs. Using a metagenomics approach Cuadrat et al. (2015) searched for NRPSs in marine samples extracted from an environment affected by upwelling in Brazil, discovering 46 condensation domains of NRPS [15]. At Lake Stechlin in north-eastern Germany, 18 NRPS clusters were identified using metagenomics data which were analyzed using antiSMASH and NAPDOS workflows [16]. An interesting result of the use of the metagenomic approach for the search of NRPs was reported by Wei et al. (2018) when analyzing samples of marine sediments from the Yellow Sea in China. NRPs diversity was evaluated based on the diversity of gene fragments from NRPS adenylation (A) domain, finding that the genes of A domain were very abundant, while the fragments of ketosynthase domain (KS) genes of type I polyketide synthase (PKS) were less abundant, suggesting that the marine sediment might have more NRPS gene clusters than PKS gene clusters distributed in this environment [17]. Given the potential of coastal areas as a source for the discovery of new molecules of commercial interest, in this work was carried out an analysis of sediment of the coast of Yucatan in order to assess the potential of microbial communities to present bacteria and genes involved in the synthesis of NRPs in two sites: one with the presence of anthropogenic contamination and the other an ecological conservation site. Through sequencing the amplicon of the 16S rRNA gene we identify bacterial species with the potential to synthesize NRPs such as antibiotics or siderophores, as well as identify metabolic capacities in microbial communities in coastal areas to produce it. In addition, a metatranscriptomic analysis was carried out to identify genes of catalytic domains present in the NRPSs and the evaluation of their transcriptional expression. Our results from the metataxonomic and metatranscriptomic analyzes showed that the phyla with the highest abundance of bacteria producing NRPs and catalytic domains of NRPSs were Proteobacteria and Firmicutes. It is important to highlight the identification of metabolic profiles and synthesis domains of NRPs in organisms of the Archaea domain. This is the first prospecting study that used high-throughput sequencing technologies that were carried out in the Yucatan Peninsula for the identification of NRPs producing bacteria and NRPSs enzymes, that demonstrates the great potential that the area represents.

## 2. Results

### 2.1. Composition of Bacterial Communities in Yucatan Wetlands

**Figure 1:**
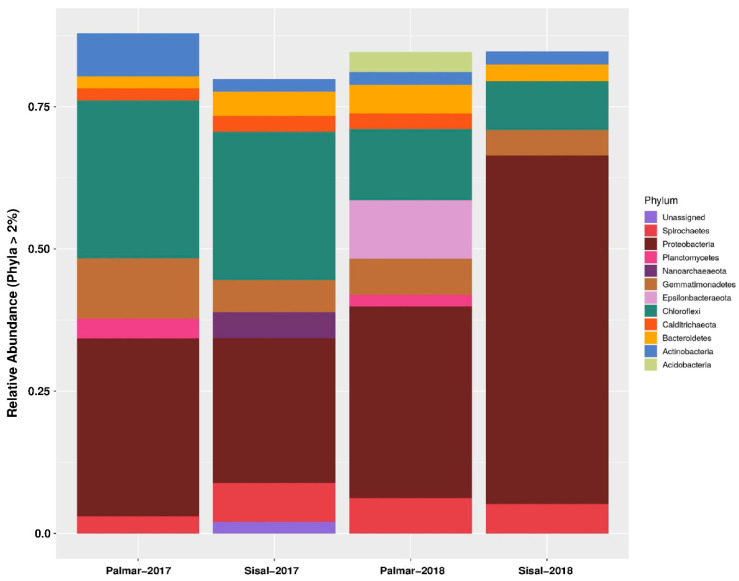
Relative abundance of bacteria composition across the two wetland sampling sites at the phylum level. The phyla represented have at least 2% relative abundance. Sisal: contaminated site; Palmar: conserved site. X-axis: sampled sites and year. Y-axis: relative abundance of phylum.

Information on the composition of the microbial community was obtained through the taxonomic assignment of the sequences obtained from the amplicon sequencing of 16S rRNA gene fragments using the Qiime2 software [18] and the Silva RNA database [19]. At the domain level, sequences were mostly assigned to Bacteria, which was numerically dominant in the two samples taken in 2017 and 2018. While the representation of the Archaea domain was low both in the two sites and in the two years of sampling. The proportion of unassigned sequences reached a maximum of 1.24% and 0.84% and was for the Sisal sample data, both for 2017 and 2018, respectively. At 99% similarity, taxonomic assignments resulted in the identification of 58 phyla such as Firmicutes, Proteobacteria, Nitrospirae, or Chlamydiae, among others. In addition, 130 classes, 280 orders, 396 families, and 495 genera were identified. Within the identified taxonomic groups, there were some that did not have a known cultivated representative (Supplementary Table S1). Relative abundances at the phylum level showed that about 80% of bacterial sequences were assigned to Proteobacteria, Chloroflexi, Gemmatimonadetes, Spirochaetes, Bacteroidetes, Epsilonbacteraeota, Actinobacteria, Calditrichaeota, Acidobacteria and Planctomycetes (Figure 1). Among these phyla, Proteobacteria were the most abundant, with at least a quarter of the assigned sequences in the four samples of both years. In the microbiota of the Sisal wetland in the 2018 sample, the abundance of Proteobacteria increased to 61%. While the abundance of Chloroflexi, Gemmatimonadetes, and Epsilonbacteraeota were mostly enriched in the ecological reserve of El Palmar. The only phylum of Archaea domain present was Nanoarchaeota in the 2017 Sisal sample (Figure 1). Phyla with a statistically significant relative abundance are found in Supplementary Figure S1. At the family level, the most abundant in the four samples of both years were Anaerolineaceae, Bacteroidetes, Calditrichaceae, Chromatiaceae, Desulfarculaceae, Desulfabacteraceae, Geminicoccaceae, Halobacteroidaceae, Kiloniellaceae, Nitrosococcaceae, Pirellulaceae, Spirochaetaceae, Syntrophobacteraceae, Thioalkalispiraceae, Thiovulaceae, and Vibrionaceae. Of the previous families, the one with the highest abundance was Vibrionaceae, but only in the Sisal sample of 2018 with 42.9%, while in the other three samples the abundance of this family was zero. Similarly, the Thiovulaceae family presented its maximum abundance in the Palmar sample in 2018 with 9.1%, while in the other samples its abundance was less than 0.6%. The proportion of unassigned taxa increased with a lower taxonomic classification (Supplementary Table S1).

### 2.2. Identification of Potential NRPs-Producing Bacteria in Coastal Wetlands of Yucatan

**Figure 2:**
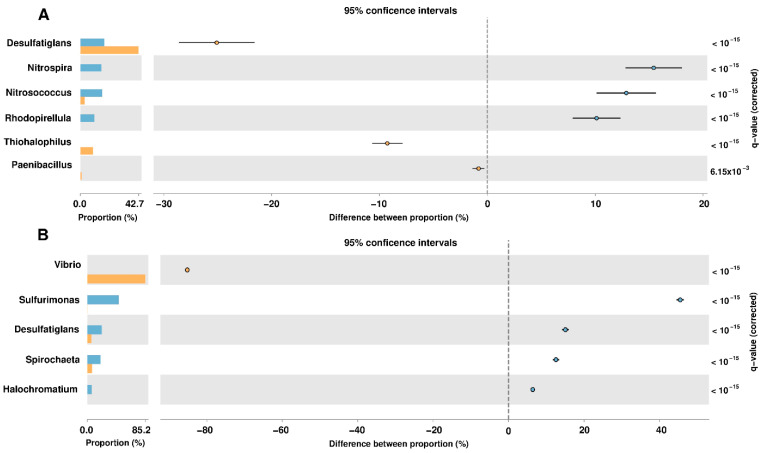
Metataxonomic profile comparisons at the genus level between the Sisal and Palmar samples using STAMP software. (**A**) The most abundant genera are shown in samples taken in 2017. (**B**) Analysis at the genus level in samples taken in 2018. A negative difference between proportions denotes a greater abundance in the Sisal group (bar and circle orange), whereas a positive difference between proportions shows a greater abundance in the Palmar group (bar and circle blue) for the given genus. Corrected *p*-values (*q*-values) were calculated based on Fisher’s exact test using Benjamini-Hochberg False Discovery Rate (FDR) correction. Features with *q* < 0.05 and the difference between proportions >2% were those that were considered biologically significant.

For the identification of taxonomic groups that were significantly represented in the samples of the analyzed sites, a statistical analysis was performed using the Statistical Analysis of Metagenomic Profiles (STAMP) [20] software. This analysis was performed using the taxonomic levels of genus, considering as biologically significant those results with a *q*-value < 0.05 (corrected p-value) and with a difference of at least 2% between the proportions of the abundance of the phylotypes of each analyzed site (Figure 2). The identification of NRPs producing bacteria in the coastal wetlands of Yucatan was carried out in the taxonomic groups obtained from the statistical evaluations. This was done by identifying the bacteria that have been reported in the scientific literature as producers of non-ribosomal peptides in our selected species and genera (Table 1).

Only two species of NRPs producing bacteria were identified in our samples of the wetlands of Yucatan coast: *Paenibacillus polymyxa* and *Vibrio vulnificus.* The genus *Paenibacillus* comprises bacterial species that produce a variety of antimicrobials, it is a cosmopolitan and ubiquitous genus that is found naturally in the soil and marine sediments [21]. Non-ribosomal lipopeptides such as polymyxins and fusaricidines were first isolated from *P*. *polymyxa* strains [22]. Polymyxins is a family consisting of a cyclic heptapeptide with a tripeptide side-chain acylated by an N-terminal fatty acid, which includes polymyxins A, B, D, E (colistin) and M (mattacin). The amphipathic property of polymyxins is essential for its antibacterial activity, which is carried out by binding to the lipid component A of lipopolysaccharide on the outer membrane of gram-negative bacteria and its subsequent breaking [23,24,25,26,27]. Polymyxin B and E are produced industrially from *P. polymyxa* strains and are used in antibiotic creams such as Neosporin, for the treatment and prevention of topical skin infections [22,27]. Others FDA-approved polymyxin B drugs include Pediotic^®^, Polysporin^®^ and Polytrim^®^ [26]. Fusaricidines were first reported in *P*. *polymyxa* KT-8 in 1996 and have been classified into four A-D families. They are non-cationic cyclic lipodepsipeptides composed of guanidinylated beta-hydroxy fatty acids bound to a cyclic hexapeptide [28,29,30,31]. This antibiotic exhibits activity against Gram-positive bacteria such as *Staphylococcus aureus* and *Micrococcus luteus* IFO 3333, through interaction with the phospholipids of cell membranes, but have weak activity against gram-negative bacteria [28,30,32]. The *V*. *vulnificus* species had an abundance of 24% in the 2018 sample obtained from the Sisal wetland. This bacterium is an opportunistic human pathogen that is found naturally in marine and estuarine environments, and in sites that have been altered by anthropogenic activities [33,34,35,36,37,38] such as the Sisal wetland. *V*. *vulnificus* produces the siderophore vulnibactin, which has a linear skeleton of norspermidine with the iron-binding moieties formed by a 2,3-dihydroxybenzoic acid residue (DHBA). This siderophore is used for the acquisition of iron that is found in limited concentrations in marine environments or during colonization of a host [39,40,41]. At the taxonomic level of the genus, different groups such as *Nitrosococcus*, *Rhodopirellula*, and *Haliangium* were found in the samples obtained from the Yucatan wetlands, which have been reported as producers of NRPs. Members of the *Nitrosococcus* genus are aerobic ammonia-oxidizing marine bacteria (AOB) such as *Nitrosococcus* *halophilus*, which encoding the genes of NRPSs for biosynthesis of amphibactin. Amphibactin is an amphiphilic siderophore consisting of a peptide group with three ornithine residues and a serine residue with a fatty acid side chain of variable structure [42]. This siderophore confers a specific competitive advantage on microbes that inhabit marine environments [43,44]. *Rhodopirellula* genus is part of the Planctomycetes phylum, whose members are recognized as producers of bioactive compounds since they share characteristics with the bioactive bacteria of the phylum Actinobacteria. In this genus, the production of NRPs has been identified in two species: *Rhodopirellula* *baltica and Rhodopirellula rubra*. *R.* *baltica* is a marine, aerobic and heterotrophic bacterium that codes for two small NRPSs, and one bimodular NRPS-PKS [45,46]. In the case of the *R*. *rubra* UC9 strain, an *in* *silico* analysis found genes related to the secondary pathways of metabolites involved in the production of bacitracin [47]. Myxobacterias are gram-negative bacteria [48,49,50] found ubiquitously in soil, but after 2005 several species of halotolerant and even obligate marine myxobacteria have been described, such as genus *Haliangium* [51], which is present in our samples from El Palmar. Within this genus, the bacterium *Haliangium ochraceum* synthesizes haliamide, a polyketide-nonribosomal peptide hybrid [52,53]. A genome analysis of *H. ochraceum* with bioinformatics tools, revealed the presence of three NRPSs and four ribosomal peptides [54], which demonstrates its potential as a source of new secondary metabolites.

### 2.3. Metabolic Pathways Prediction of NRPs Production

**Figure 3:**
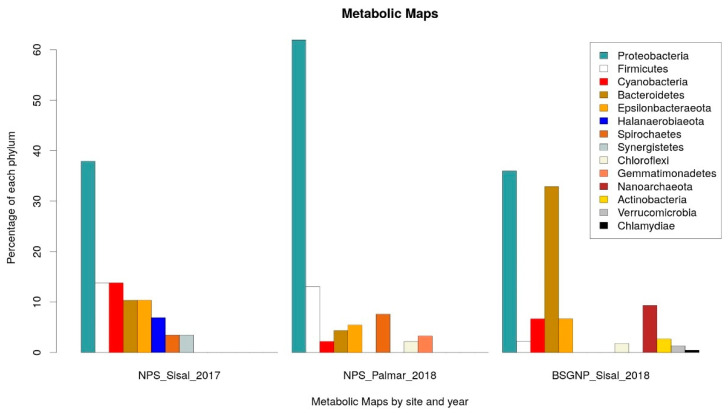
Phyla associated with the metabolic Kyoto Encyclopedia of Genes and Genomes (KEGG) maps of NRPs. On the left: Phyla associated with the Nonribosomal Peptide Structures (NPS) map enriched in the 2017 Sisal sample. In the middle: Phyla associated with the NPS map enriched in the 2018 El Palmar sample. On the right: Phyla associated with the Biosynthesis of siderophore group nonribosomal peptides (BSGNP) map enriched in the 2018 Sisal sample.

For the identification of potential pathways associated with the production of NRPs in the microbial communities of the wetlands of the Yucatan coast, we carried out the prediction of the metabolic capacities of the bacteria identified in the samples taken in Sisal and El Palmar. The analysis was performed using the PICRUSt2 [55,56] software to predict the metabolic capabilities of the identified bacteria, and Kyoto Encyclopedia of Genes and Genomes (KEGG) [57] database to functional annotation at level 3: specific pathway associated with a specific function. The identification of the statistically significant pathways was performed using the STAMP software, establishing as significant those molecular functions that had a *q*-value < 0.05 and a difference in the proportion of annotated sequences of 0.5. We focus only on those pathways that had some direct relationship with the production of NRPs. For the data obtained in 2017, the production of Nonribosomal Peptide Structures was enriched in the sample of Sisal wetland. From the analysis carried out with PICRUSt2, the proportion of taxonomic groups identified at the phylum level associated with each metabolic pathway was calculated (Supplementary Tables S2 and S3). It was observed that the three main phyla associated with the production of Nonribosomal Peptide Structures in the 2017 data were Proteobacteria, Firmicutes and Cyanobacteria (Figure 3). In the case of the samples obtained in 2018, the Biosynthesis of Siderophore Group Nonribosomal Peptides (BSGNP) was the metabolic route enriched in Sisal; the three phyla with the highest proportion were Proteobacteria again and Bacteroidetes of the Bacteria domain, and Nanoarchaeota of the Archaea domain (Figure 3).

In the functional predictions for El Palmar data of 2018, the enriched metabolic pathway was that of Nonribosomal Peptide Structures (NPS) (Figure 3). The phyla Proteobacteria and Firmicutes were what had a greater proportion of phylogroups (Figure 3), as was observed in the Sisal sample. The only notable change is that the third most abundant group was the Spirochaetas, which were the least abundant on this pathway in the 2017 Sisal sample.

### 2.4. Metatrancriptomics Identification of NRPSs

**Figure 4:**
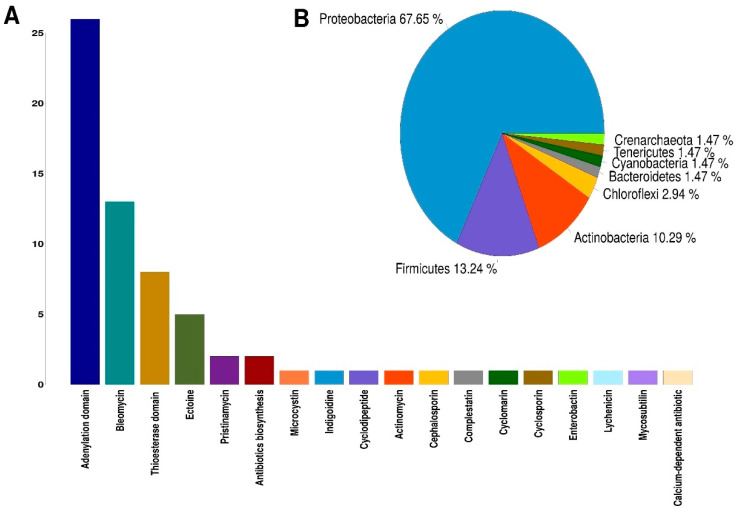
Catalytic domains of nonribosomal peptide synthetases (NRPSs) identified in our metatranscriptomic data. (**A**) Catalytic domains of NRPSs and their products identified in metatranscriptomic data from Sisal and Palmar samples. (**B**) Phyla in which the presence of the catalytic domains present in the NRPSs were detected. The number represents the percentage of NRPS’s sequences in each phylum.

On average, 58 million raw sequences were obtained from the metatranscriptome from each analyzed site, and after having removed the adapters and having performed quality filtering, an average of 49 million high-quality sequences were obtained. A total of 109,478 genes were identified after de novo assembly using Trinity [58] program. Using the BLASTX [59] program and the UniProtKB/Swiss-Prot [60] database, 46,776 genes were identified that encode a protein already described. To carry out the identification of NRPSs, we used the signature Hidden Markov Models (HMMs) for biosynthetic genes reported by Blin et al. (2012) [61]. First, using the PFAM [62] database and the HMMER [63] program, the identification of the functional domains present in the proteins obtained from the sequences assembled by the Trinity program was carried out. After, the sequences that presented the NRP biosynthesis profiles reported by Blin et al. (2012) and with a threshold of e-value < 10^−3^ were selected. In this way, 45 sequences associated with signatures HMMs for the biosynthesis of NRPs were obtained (Supplementary Tables S4 and S5). A second round was conducted to find NRPSs using the NaPDoS [64] web tool, which identifies the condensation domains and their possible products. This search resulted in the identification of 23 sequences that were selected for having a coincidence of at least 80% identity against the condensation domains of the NaPDoS database (Supplementary Tables S4 and S5). The quantification of the expression levels of the sequences identified as NRPSs was performed through the differential expression analysis of metatranscriptome data using the DESeq2 [65] program. Of the four catalytic domains found in most NRPSs, three were identified in this work: adenylation (A) domain; condensation (C) domain and thioesterase (TE) domain. The condensation domain was the most abundant, identifying 25 sequences in Palmar, eight sequences in Sisal, and 1 sequence that does not have a significant expression in either of the two sampled sites (Supplementary Tables S4 and S5). The adenylation domain was the second most abundant, with 20 sequences in the Sisal sample and six sequences in the Palmar sample (Table 2). For the thioesterase domain, eight sequences were identified, four in Sisal and four in Palmar (Table 2). The products synthesized mainly by the condensation domains identified were antibiotics, such as bleomycin, pristinamycin, actinomycin, cephalosporin, among others (Figure 4A). The second abundant product synthesized by the condensation domains was ectoine, for which we identified five sequences (Figure 4A). Ectoine is a widely distributed solute in different halophilic and halotolerant microorganisms, produced in response to osmotic stress and functions as a potent protector of osmostress [66,67]. Ectoine improve protein folding and protects biomolecules such as enzymes, nucleic acids, and even whole cells against various stress conditions [68]. These multifunctional effects have fostered the development of a wide range of skincare and dermatological cosmetic preparations as moisturizers in cosmetics for the care of aged, dry or irritated skin [69,70]. Other products synthesized by the condensation domain that were identified in a smaller proportion were the indigoidine, which is a natural blue pigment with potential applications in the dye industry [71]. The enterobactin siderophore that mediates iron absorption and that is selectively imported into bacteria; which is used to generate siderophores conjugates as a promising strategy for the detection of bacteria or to enhanced antibacterial activity of antibiotics by supplying functional reagents for Trojan-horse-type delivery [72,73,74]. The antifungal lipopeptide mycosubtilin, which is used for the biocontrol of the *Pythium* *aphanidermatum* pathogen in tomato seedlings [75]. Cyclomarin, which is a highly potent antimycobacterial and antiplasmodial cyclopeptides [76] (Figure 4A).

The taxonomic group with the highest number of catalytic domains identified were Proteobacteria with almost 68% of the sequences, followed by Firmicutes with 13% of sequences identified. Actinobacteria only had 10% of sequences identified (Figure 4B). These results agree with those found in metabolic analysis, where Proteobacteria and Firmicutes were the phyla with the highest presence of metabolic pathways associated with the production of NRPs. It should be noted that the condensation domain that produces the cyclomarin molecule is present in an organism that was identified as belonging to the Crenarchaeota phylum, which is an Archaea.

## 3. Discussion

Actinobacteria have been described as the largest producers of NRPs, producing compounds such as antibiotics, siderophores or biosurfactants, among others [77]. Most of Actinobacteria were isolated from terrestrial environments [17]. Our metataxonomic analysis of the sediments of the wetlands of the Yucatan coast showed that the identified bacteria that have a greater representation belong to the phyla Proteobacteria, Chloroflexi, Gemmatimonadetes, Spirochaetes, and Epsilonbacteraeota. Actinobacteria were underrepresented in our studied samples, which is consistent with that reported by Wei et al. (2018) of the Yellow Sea sediments [17] (Figure 1). When marine environments are explored for search potential NRPs-producing bacteria, the most abundant organisms found were Proteobacteria and Bacteroidetes, not the Actinobacteria [17]. The genera and species of bacteria identified as producers of NRPs in our study, belong to the phyla Proteobacteria and Firmicutes. The null identification of genera and species of NRPs producing bacteria belonging to the Actinobacteria phylum is due to the fact that no known cultured representatives could be identified in the analyzed data. Taxonomic annotations at the level of genus and species within the Actinobacteria came from uncultivated organisms, which can be interpreted as new phylogroups present in the coastal area of Yucatán that have not yet been described within the Actinobacteria. From the statistical analysis to evaluate the differences between the proportions of the taxonomic groups identified in the sediments of the study sites, it was observed that the presence of organisms belonging to genera and species reported as producers of NRPs is greater in the sample obtained from sediments of El Palmar. In 2017, two (*Nitrosococcus*, *Rhodopirellula*) of the five genera identified as producers of NRPs were present in El Palmar and only one (*Paenibacillus*) in the Sisal sample; while for the 2018 data, three (*Paenibacillus*, *Nitrosococcus*, *Haliangium*) of the five genera were in El Palmar and only one (*Vibrio*) in the Sisal sample. Further studies are necessary to determine if these changes in the abundance of the population of NRPs producing bacteria are due to seasonal fluctuations, or if they are determined by some other factor.

From the analysis of the metabolic profiles that were predicted for the microbial communities of the Yucatan coastal zone, we identified two metabolic maps directly associated with the production of NRPs. The first was the Nonribosomal peptide structures map identified in the sediment samples of Sisal (2017) and El Palmar (2018); and the second was the Biosynthesis of siderophore group nonribosomal peptides map identified only in Sisal sediments (2018). The phyla Proteobacteria and Firmicutes were the ones that had the greatest contribution to non-ribosomal peptide structures, both in the Sisal sample in 2017 and in the El Palmar sample in 2018. Antibiotics, such as bacitracin, tyrocidin, or gramicidin, produced by bacteria from the phylum Firmicutes [78,79] are classified within the metabolic map of noribosomal peptide structures. While organisms of the phylum Actinobacteria were not associated with the metabolic map of non-ribosomal peptide structures, although 80% of the antibiotics currently used are derived from Actinobacteria [80,81]. It seems that the environmental conditions from which the samples are obtained may be a factor that not only determines the presence of the Actinobacteria, but also the metabolic processes they carry out. In a study conducted by Parera-Valadez et al. (2019) with marine sediment samples collected between 2 and 30 m by scuba diving, isolated nine different genera of Actinomycetes, managing to identify the antibiotic resistomycin in one of its isolates [82]. While in our study and in the one carried out by [17], the abundance of Actinobacteria was low in coastal and marine sediments, respectively. Regarding the metabolic map of Biosynthesis of siderophore group nonribosomal peptides, the first three phyla that had the highest representation were Proteobacteria, Bacteroidetes and Nanoarchaeota; while Actinobacteria were in sixth place. The phylum Nanoarchaeota was the only one of Archaea domain that presented a metabolic map to produce NRPs such as siderophores. This is interesting, since little is known about the assimilation of iron in Archaea organisms in marine environments and, therefore, about the production of siderophores in these organisms. There are works such as that carried out by Dave et al. (2006) in which they have demonstrated the presence of siderophores in marine archaea isolated in India from coastal areas [83]; or that performed by Patil et al. (2016) in which they identified siderophores of the hydroxamate type from haloalkaliphilic bacteria isolated from Lonar Lake in India [84].

In order to have a more detailed picture not only of potential NRPs producing bacteria, but also of the possible gene sequences associated with the production of NRPs with an active expression at the sampling sites, a metatranscriptomic study was carried out of the microbial communities. From our metatranscriptomic data of Sisal and Palmar, we achieved the identification of 68 sequences associated with three catalytic domains present in the NRPSs enzymes: adenylation (A) domain; condensation (C) domain; and thioesterase (TH) domain. Of all sequences, 52% identified from the metatranscriptome were found in the sample obtained from El Palmar, which is consistent with our metataxonomic results where the proportion of bacteria reported as producers of NRPs was higher in said site. The condensation domain was the most abundant identified in El Palmar sample, and the antibiotics the product they synthesized mostly. The adenylation domain was the second with the highest number of sequences identified, being the Sisal site where most of them were found. In the case of the thioesterase domain, an equal number of sequences were found in both Sisal and Palmar. The phylum Proteobacteria was the one that had the highest number of catalytic domains of NRPSs, followed by Firmicutes, both having 80% of all identified sequences. While Actinobacteria only had 10% of the sequences identified as catalytic domains of NRPSs. The production of NRPs carried out by Archaea organisms was also detected from our metatranscriptomic data. The condensation domain that performs the synthesis of the antimicobacterial cyclomarin, was identified in an organism belonging to the Crenarchaeota phylum in the sample of El Palmar. This is a very prominent result since it opens the possibility to the search for new structures of NRPs, but in general, it opens the possibility to the search in Archaea for NRPs with new structures not previously described, since the identification of nonribosomal compounds has focused only in certain groups of bacteria.

## 4. Conclusions

This is the first study in which a systematic analysis of sediments of the wetlands of the Yucatan coast is carried out using next-generation sequencing tools, for the metataxonomic search of bacteria producing nonribosomal peptides. As well as for the identification of gene sequences that encode catalytic domains present in NRPSs enzymes, using a metatranscriptomic approach. From our taxonomic profiles analysis, we have observed that the abundance of bacteria that have traditionally been associated with the production of NRPs, such as Actinobacteria, is low in the coastal sediments of the wetlands of the Yucatan coast. While organisms of the phyla Proteobacteria and Firmicutes were those that presented a greater number of NRPs producing genera. This can also be observed from our metatranscriptomic data, where 80% of the sequences identified as catalytic domains of NRPSs enzymes were found in the phyla Proteobacteria and Firmicutes. While in Actinobacteria only 10% of the sequences of the catalytic domains of NRPS enzymes were identified. Similarly, the metabolic maps to NRPs production identified in the microbial communities of the two study sites are mainly associated with organisms belonging to the phyla Proteobacteria, Firmicutes, Cyanobacteria, Spirochaetas and Nanoarchaeota. The largest number of taxonomic genera producing NRPs were identified at El Palmar ecological conservation site, as well as 52% of the sequences of catalytic domains present in NRPSs. This highlights the importance of ecological reserves as sources of organisms producing secondary metabolites with great potential for biotechnological use, and the relevance of their preservation and environmental management. It is important to highlight the identification of metabolic profiles for siderophores production in Nanoarchaeota. As well as the identification of sequences of condensation domains that produce the antiplasmodial cyclomarin in organisms belonging to the Crenaracheota phylum. As traditionally the search for nonribosomal compounds focuses on bacteria, our results open the possibility to the search for new nonribosomal structures in the Archaea. More metataxonomic studies are needed to allow the inventory and location of bacteria and places of greater relevance for the search of NRPs producing organisms. In addition to metatranscriptomic studies that allow the identification of genes involved in the production of noribosomal compounds present in the Yucatan coast.

## 5. Materials and Methods

### 5.1. Site Description and Sample Processing

The comparison of the microbial communities present in the sediments of two wetlands with different degrees of anthropogenic impact in the Yucatan Peninsula, was made by selecting the contaminated site of the Sisal swamp, Yucatán (21°09’43.6” N; 90°02’27.2” W) and the conserved site of a swamp within the state ecological reserve of El Palmar (21°08’56.4” N; 90°06’07.0” W) in the state of Yucatan. Three sediment samples were taken for each site, for which three points were chosen within a box meter square: one point at the center and two more at the ends. The samples were extracted approximately 20 cm deep, and 2 g of sediment were taken from each point and mixed with 6 mL of the LifeGuard Soil Preservation buffer (Qiagen, Hilde, Germany) and stored at −20 °C. The experiment was conducted at two different times, one in May 2017 and March 2018 in the same places.

### 5.2. Nucleic Acids Extraction, Metataxonomic and Transcriptomic Sequencing

Nucleic acids extraction, libraries construction and sequencing were requested from the Research and Testing Laboratory (Lubbock, TX, USA). For DNA extraction it was used Qiagen MagAttract PowerSoil DNA KF Kit (Qiagen, Hilde, Germany) following the manufacturer’s recommendations. After quality and purity evaluation of extracted DNA, the V3–V4 region of the 16S ribosomal DNA (rDNA) gene was amplified used the bacteria-specific primer pair 357wF (5′-CCTACGGGNGGCWGCAG-3′) and 785R (5′-GACTACHVGGGTATCTAATCC-3′). Amplifications were performed in 25 µL reactions with Qiagen HotStar Taq master mix (Qiagen Inc, Valencia, California), 1 µL of each 5 μM primer, and 1ul of template, reactions were performed on ABI Veriti thermocycler (Applied Biosytems, Carlsbad, CA, USA). Amplification products were visualized with eGels (Life Technologies, Grand Island, New York, NY, USA). Products were then pooled equimolar and each pool was size selected in two rounds using SPRIselect Reagent (BeckmanCoulter, Indianapolis, IN, USA) in a 0.75 ratio for both rounds. Size selected pools were then quantified using the Qubit 4 Fluorometer (Life Technologies) and loaded on an Illumina MiSeq (Illumina, Inc. San Diego, CA, USA) 2 × 300 flow cell at 10 pM. For RNA extraction, only the samples taken during the month of March 2018 were used, for which Qiagen RNeasy PowerSoil Total RNA Kit (Qiagen, Hilde, Germany) was used following the manufacturer’s recommendations. RNA sequencing (RNA seq) libraries were constructed and sequenced following a default Illumina stranded RNA protocol. Sequencing was done using an Illumina HiSeq 2500 platform to generate 2 × 150 bp paired-end reads. Sequencing resulted in an average yield of 58 million reads per sample.

### 5.3. Metataxonomic Data Analysis

The sequences obtained from the three sampled points of each site were joined to form a single set of data per analyzed place. Thus, in the end, we left with a single data set for Sisal and another for El Palmar in 2017, and the same for the 2018 sampling. The analysis was carried out on four data sets, two for Sisal 2017–2018 and two for El Palmar 2017–2018. Data processing was performed using Quantitative Insights into Microbial Ecology 2 (QIIME2) [18]. Paired-end sequences were imported into QIIME2 and DADA2 [85] was used to perform PhiX sequence filtering, chimera sequence elimination and sequence variant (SVs) detection. The forward sequences were truncated to 285 base pairs because the quality of reads after base 285 declined, and to reverse sequences were truncated to 201 base pairs for the same reason. The taxonomic classification of SVs was performed using a Naive Bayes fitted classifier, trained at 99% identity with Silva 132 QIIME-compatible database [19] for the Forward/Reverse primer set. A series of alpha and beta diversity indices were calculated using the phyloseq package [86] implemented in R, such as rarefaction curves, Chao estimator, Shannon index, and principal coordinate analysis (PCoA) using the UniFrac distance matrix [87] weighted.

Functional profiles of microbial communities were predicted by Phylogenetic Investigation of Communities by Reconstruction of Unobserved States version 2 (PICRUSt2) [55,56] from observed data of the taxa identified using the 16S rDNA reads analyzed with QIIME2. Functional predictions were assigned up to all KEGG [57] orthology (KO) numbers, to obtained KEGG pathway abundance information. The taxonomic groups identified, as well as the predicted metabolic functions for the microbial communities were statistically analyzed to assess the existence of significant differences in their relative proportions. To carry out the statistical evaluation we using the Statistical Analysis of Metagenomic Profiles (STAMP) software v2.1.3 [20]. A Fisher’s exact test was implemented for hypothesis testing and Benjamini-Hochberg False Discovery Rate (FDR) correction was applied on these data to identify statistically significant differential features among metagenomes. Results with *q*-value < 0.05 (corrected *p*-value) were considered as significant and the biological relevance of the taxonomic statistics was determined by applying a difference of at least 2% between the proportions; in the case of metabolic functions, the biological relevance of predictions were determined by applying a difference of at least 0.5% between the proportions.

### 5.4. Metatranscriptomics Data Analysis

The raw data obtained from the sequencing by RNA-seq of the triplicates for each site were first filtered to remove adapters, as well as low-quality reads, using the NGS QC Toolkit v2.3.3 software [88], and its program IlluQC.pl for Ilumina data using default parameters. A library of Palmar samples was removed due to the low number of reads obtained, staying at the end with 5 libraries: 2 for El Palmar site, and 3 for Sisal site. Subsequently, the filtered reads were assembled by de novo assembly package Trinity [58]. Annotation of assembled sequences was performed locally using BLASTX [59] sequence similarity searches against the protein UniProtKB/Swiss-Prot [60] database, with a threshold of e-value < 10^−3^. Identification of non-ribosomal peptide synthetases (NRPSs) was carried out through the use of HMMs profiles of the antiSMASH database reported by Blin et al. [61], and the Natural Product Domain Seeker (NaPDoS) bioinformatics tool [64]. First, in the proteins obtained by Trinity from the assembled sequences, functional proteic domains were identified using locally the HMMER [63] sequence analysis program and the HMM profiles of the PFAM [62] protein database, with a cut-off of e-value < 10^−3^. Then, in our sequences annotated in the previous step, we identified those that presented the signature HMMs for the detection of secondary metabolite biosynthesis genes reported by Blin et al. [61] corresponding to NRPSs and that also had a threshold of e-value < 10^−3^. For the detection and analysis of the condensation (C) domains present in the NRPSs, the sequences assembled by Trinity were sent to the NaPDoS web tool. The results obtained were filtered to keep those sequences that had hits of at least 80% identity against the condensation domains of the NaPDoS database. The DESeq2 [65] package was used to normalize the reads data and for the negative binomial statistical test of the gene expression in the samples, with the aim of knowing in which of the two sampling sites there is a greater abundance of transcripts identified as NRPSs.

## Figures and Tables

**Table 1:** Genera and species present in coastal wetlands of Yucatan with the presence of nonribosomal peptides (NRPs) described in the literature.

Genus (Specie)	NRP	Type of Metabolite	2017	2018
*Paenibacillus *(*P. polymyxa*)	Polymyxins; Fusaricidins	Antibiotics	Sisal *	Palmar *
*Vibrio* (*V. vulnificus*)	Vulnibactin	Siderophore	-	Sisal
*Nitrosococcus*	Amphibactin	Siderophore	Palmar	Palmar *
*Rhodopirellula*	Bacitracin	Antibiotic	Pamar	-
*Haliangium*	Haliamide	Cytotoxic	-	Palmar *

The last 2 columns show the places where there was a statistically significant enrichment of the NPRs producing organisms. The differences between the proportions of abundance were ≥2%, except for those indicated by *, which presented a difference between proportions ≥0.5%. Sisal: contaminated site. Palmar: preserved site.

**Table 2:** The top 5 catalytic domains, their Hidden Markov Models signatures and the predicted products identified in the metatranscriptome of Sisal and El Palmar samples. The number of sequences identified in each sample was quantified using DESeq2 program.

Description	PFAM ID/Product	Number of Sequences in Sisal	Number of Sequences in Palmar
Adenylation domain	PF00501	20	6
Condensation domain	Bleomycin	0	13
Thioesterase domain	PF00975	4	4
Condensation domain	PF06339. Ectoine	3	1
Condensation domain	Pristinamycin	0	2

## Supplementary Materials

The following are available online at https://www.mdpi.com/2072-6651/12/6/349/s1, Table S1: Reads assigned to bacterial OTUs, Table S2: Bacterial OTUs assigned to Metabolic KEGG maps 2017, Table S3: Bacterial OTUs assigned to Metabolic KEGG maps 2018, Table S4: Identified catalytic domains of NRPSs enzymes, Table S5: Sequences identified as catalytic domains of NRPSs in the fasta format, Figure S1: Metataxonomic profile comparisons at the phylum level between the Sisal and Palmar samples using STAMP software.
