# Supplementary material for: Mining the Yucatan Coastal Microbiome for the Identification of Non-Ribosomal Peptides Synthetase (NRPS) Genes
Source: Toxins (Basel). 2020 May 26;12(6):349. doi: 10.3390/toxins12060349 (PMC7354552; doi:10.3390/toxins12060349)
Supplement: Supplementary file 1 [file toxins-12-00349-s001.zip › toxins-739815 - Supplem..pdf]

Supplementary Materials: Mining the Yucatan Coastal Microbiome for the Identification of Non-Ribosomal Peptides Synthetase (NRPS) Genes

Mario Alberto Martínez-Núñez and Zuemy Rodríguez-Escamilla

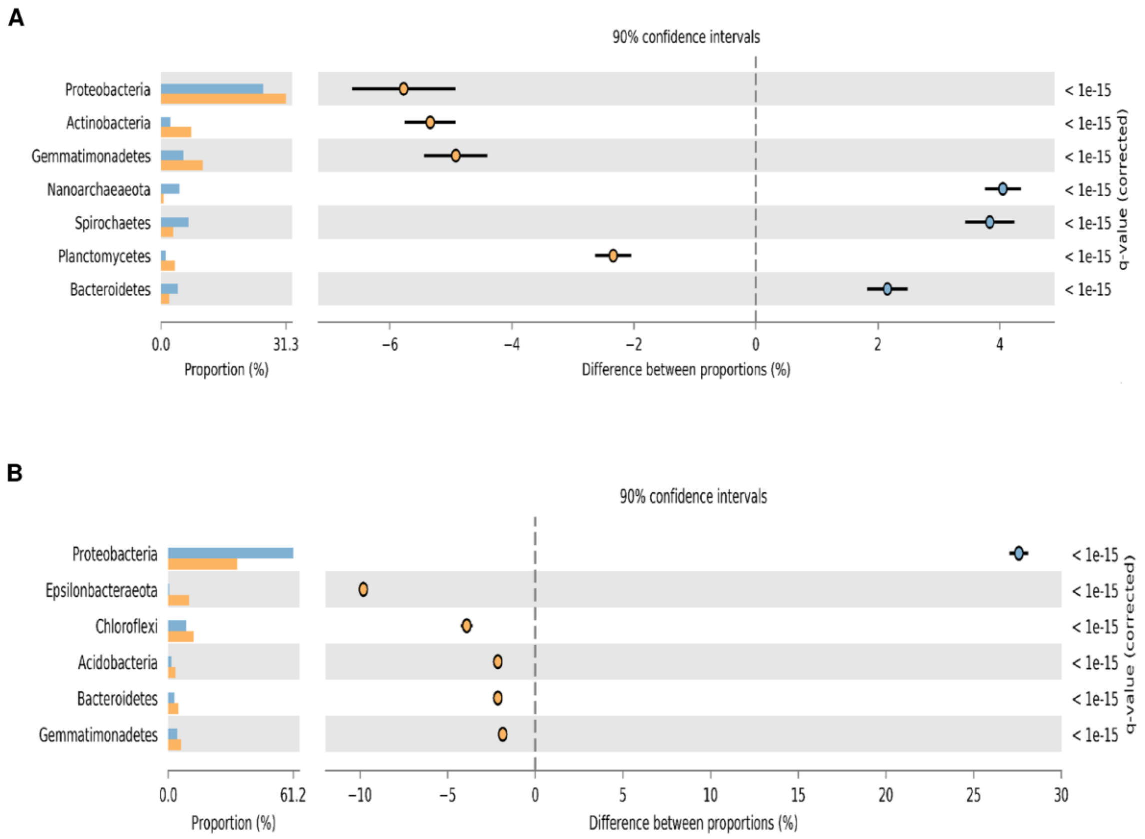

Figure S1. Metataxonomic profile comparisons at the phylum level between the Sisal and Palmar samples using STAMP software.
